# Association between Physical Activity, Body Composition, and Metabolic Disorders in Middle-Aged Women of Ksar el Kebir (Morocco)

**DOI:** 10.3390/ijerph20031739

**Published:** 2023-01-18

**Authors:** Khouloud Harraqui, Dia Eddine Oudghiri, Hanae Naceiri Mrabti, Zineb Hannoun, Learn-Han Lee, Hamza Assaggaf, Ahmed Qasem, Khang Wen Goh, Long Chiau Ming, Ching Siang Tan, Abdelhakim Bouyahya, Abdellatif Bour

**Affiliations:** 1 Laboratory of Biology and Health (LBS), Nutrition, Food and Health Sciences Team, Faculty of Sciences, Ibn Tofail University, Kenitra 14000, Morocco; 2 Biology and Health UAE/U23FS Team, Department of Biology, Faculty of Sciences, Abdelmalek Essaâdi University, Avenue de Sebta, Mhannech II, Tetouan 93002, Morocco; 3 High Institute of Nursing Professions and Health Techniques Casablanca, Casablanca 20250, Morocco; 4 Novel Bacteria and Drug Discovery Research Group (NBDD), Microbiome and Bioresource Research Strength (MBRS), Jeffrey Cheah School of Medicine and Health Sciences, Monash University Malaysia, Sunway City 47500, Malaysia; 5 Department of Laboratory Medicine, Faculty of Applied Medical Sciences, Umm Al-Qura University, Makkah 21955, Saudi Arabia; 6 Faculty of Data Science and Information Technology, INTI International University, Nilai 71800, Malaysia; 7 School of Medical and Life Sciences, Sunway University, Sunway City 47500, Malaysia; 8 School of Pharmacy, KPJ Healthcare University College, Nilai 71800, Malaysia; 9 Laboratory of Human Pathologies Biology, Department of Biology, Faculty of Sciences, Mohammed V University in Rabat, Rabat 10100, Morocco

**Keywords:** perimenopause, post-menopause, anthropometry, metabolic syndrome, dyslipidemia, diabetes, IPAQ, physical activity

## Abstract

This study aimed to examine the association between physical activity (PA), body composition, and metabolic disorders in a population of Moroccan women classified by menopausal status. This cross-sectional study comprised 373 peri- and postmenopausal women aged 45–64 years old. PA levels were assessed using the short version of the International Physical Activity Questionnaire (IPAQ-SF). Body composition and metabolic disorders were assessed by measurements of anthropometric and biological parameters: weight, body mass index (BMI), waist circumference (WC), hip circumference (HC), WC/HC ratio, percent body fat, systolic and diastolic blood pressure, fasting blood glucose, and serum lipids (total cholesterol (TC), triglycerides (TG), HDL-C, and LDL-C). Metabolic syndrome (MetS) was diagnosed according to the National Cholesterol Education Program Adult Treatment Panel III (NCEP-ATP III) criteria. Pearson correlations were used to test for associations. The mean total PA score of perimenopausal women was 1683.51 ± 805.36 MET-min/week, and of postmenopausal women was 1450.81 ± 780.67 MET-min/week. In all participants, peri- and postmenopausal women, PA was significantly and inversely associated with BMI, weight, percent body fat, HC, WC, and number of MetS components (*p* < 0.01), and with fasting blood glucose, TC, TG, and LDL-C (*p* < 0.05). The frequencies of metabolic disorders, obesity, abdominal obesity, type 2 diabetes, dyslipidemia, and MetS were significantly lower at moderate and intense levels of PA (*p* < 0.05), in also all participants. In middle-aged women, particularly those who are peri-menopausal, PA at moderate and intense levels is associated with more favorable body composition and less frequent metabolic disorders. However, in this particular study, PA does not appear to be associated with blood pressure and HDL-C concentrations. Future studies may be needed to further clarify these findings.

## 1. Introduction

Middle-aged women experience social, physical, and psychological changes that can influence their quality of life and overall health [1]. Menopause affects the lives of all women and corresponds to a physiological phase caused by the cessation of ovarian activity [2]. This phase generally includes the perimenopausal period where changes in estrogen levels lead to irregular menstrual periods marking the end of the childbearing years [3], and the postmenopausal period, which occurs after 12 months of amenorrhea following the last period [4].

During menopause, hormonal changes, particularly the decrease in estrogen, contribute to changes in body composition [5], and, specifically, increased body weight [6], redistribution, and increase in body fat [7], increase in abdominal fat [7], and decrease in muscle mass [8]. This estrogen decline is also associated with the emergence of numerous metabolic disorders and an increased risk of cardiovascular complications in postmenopausal women [9,10]. Metabolic disorders can be defined as alterations or abnormalities that occur as a result of disruptions, alterations, or dysfunction of normal metabolic processes in the body [11,12]. Metabolic disorders, such as obesity, abdominal obesity, dyslipidemia, hypertension, type 2 diabetes, and impaired fasting glucose and insulin resistance, may constitute the components of the metabolic syndrome, and are most frequent during menopause [13,14]. Indeed, the changes that come with menopause can affect the quality of life of women and become severe in those who do not engage in physical activity [13].

Physical activity (PA) provides health benefits for menopausal women [15]. This activity is considered an effective means of preventing and significantly reducing metabolic disorders [16], including obesity, diabetes, altered serum lipids, high blood pressure, and metabolic syndrome [17,18]. It has been proposed as a treatment option for the risk of metabolic disorders in postmenopausal women [19]. In addition, studies have reported that regular PA is associated with improved body composition in menopausal women, such as control of a healthy weight, prevention against progressive weight gain [6,20], reduction in total and abdominal fat mass [21], and decreased bone loss [22]. On the other hand, physical inactivity has been considered the fourth leading risk factor for mortality in the world, causing about 3.2 million deaths globally. It is also the cause of about 27% of diabetes cases and 30% of heart disease cases [16]. In this regard, reducing sedentary lifestyle, adopting an active lifestyle, and performing regular PA are essential and recommended to mitigate and prevent menopausal disorders, and to ensure good health in postmenopausal women [23].

In Morocco, the nutritional and epidemiological transition characterized by changes in dietary habits (deviation from the Mediterranean diet model) and lifestyle, including sedentary mode and decreased physical activity, contribute to the increase in diseases and/or metabolic disorders, as indicated above, and mainly in the adult population of urban women [24,25]. According to the results of the National Survey on Common Risk Factors for Noncommunicable Diseases (STEPS) conducted by the Ministry of Health in 2017–2018 on the population aged 18 years and older, the rate of lack of PA was estimated at 21.1% (higher in women (26%) than in men (16.1%)). The prevalence of overweight, obesity, diabetes, and hypertension were 33%, 20%, 10.6%, and 29.3%, respectively [26].

However, today there is still a lack of studies on PA in Morocco, and, in particular, on menopausal women in the northern region, hence the interest of our research is to provide new results that can serve as data for the literature, and that will be useful in a mapping of this subject throughout the kingdom. Studies have assessed the cardiorespiratory fitness of Moroccan women (45–65 years old) using fitness tests and reported that women had better cardiorespiratory fitness [27,28]. Indeed, to our knowledge, no study has been conducted to assess the relationship between PA levels and anthropometric and biological variables in peri- and postmenopausal women in the northern region. The objective of this study is to assess PA levels and to investigate the association between physical activity, body composition, and metabolic disorders in a sample of women from the city of Ksar el Kebir, classified as perimenopausal and post-menopausal. We hypothesized that PA would be associated with a favorable body composition and less frequent metabolic disorders in both groups of women, but mainly in the perimenopausal group.

## 2. Materials and Methods

### 2.1. Study Design

This epidemiological study was conducted from January 2019 to February 2020, on a population of healthy women, aged 45–64 years, and residing in the city of Ksar el kebir in Morocco.

After obtaining an authorization signed by the regional delegate of the Ministry of Health and the director of the hospital in Ksar el kebir. Middle-aged women who consulted the health centers and the hospital in the city of Ksar el Kebir for a medical examination or for any other reason were invited to participate in the study. Of the invited women who voluntarily agreed to participate in this study, those who met the criteria were informed of the purpose and procedures of the study through a detailed description of the protocol to be followed to collect the data. All study participants signed and provided their informed consent forms before beginning the survey. Confidentiality, anonymity, and the possibility to refuse to participate, or to withdraw from the study were respected. The study was conducted in accordance with the Declaration of Helsinki (WORLD MEDICAL ASSOCIATION, 2008) [29].

The study was conducted in the hospital of the city of Ksar el kebir. The participants’ body composition and metabolic disorders were assessed by measuring anthropometric and biological parameters (weight, BMI, WC, HC, WHR, percent body fat, systolic and diastolic blood pressure, and biochemical analyses of fasting blood glucose and serum lipids), which together constitute the markers of body composition and metabolic disorders. PA was measured using the International PA Questionnaire (IPAQ-SF).

### 2.2. Study Participants

The study sample included 373 healthy participants. The participants were divided into two groups according to their menopausal status, a group of perimenopausal women (*n* = 160) with a mean age of 48.84 ± 2.4 years, and a group of postmenopausal women (*n* = 213) with a mean age of 56.65 ± 4.29 years [30].

The inclusion criteria for this study were: age 45–64 years, menopausal status (perimenopausal or post-menopausal), Moroccan nationality, residence exclusively in Ksar el kebir, and signed informed consent from each participant. The exclusion criteria were: pregnancy or breastfeeding, surgical menopause, taking hormonal treatments, taking medication that could affect the results, and the presence of a serious chronic disease. Participants who did not meet the inclusion criteria and who had missing data were also excluded from the study.

### 2.3. Data Collection and Instrumentation

Recruitment and data collection were conducted in the department of General Medicine of the hospital-Ksar el kebir, by a team composed of a nutritionist, a nurse, a doctor of the department, and me. Face-to-face interviews were conducted in a private room to collect information from the participants, fill in the PA level assessment questionnaire (IPAQ), take anthropometric measurements and blood samples for biochemical analysis. All participants were instructed on how to answer the questions during the survey. For illiterate participants, the team took the time to clearly explain each question and how to answer it, point by point, in a simple manner adapted to each participant, and their level of understanding, so that the questions were clear, and the participants understood until they easily gave their own answers at the end, allowing the team to record their information and fill in the questionnaire accordingly.

#### 2.3.1. Menopausal Status

Menopausal status was determined with a self-reported menstrual diary. Participants answered questions based on their menstrual status, including date of onset of menstrual irregularity and date of last menstrual period. Participants’ date of birth was recorded. Based on their own responses, participants were classified as perimenopausal (women reporting irregularity or cessation of menses for less than one year) and postmenopausal (women reporting amenorrhea for at least 12 months) [31].

#### 2.3.2. Anthropometric Measurements

All measurements were taken with standard procedures, by the research team. Participants were shoeless, in light clothing, and with an empty bladder. Height was measured in the standing position by a portable stadiometer (SECA 217, accuracy 0.1 cm). Weight was measured on a mechanical scale (SECA 762, accuracy 0.1 kg). Body mass index (BMI) was calculated by dividing weight by height squared, expressed in Kg/m^2^. Waist circumference (WC) and hip circumference (HC) were measured in underwear by a tape measure (SECA 201, accuracy 0.1 cm). Waist circumference was measured on the diameter of the midpoint between the iliac crest and the lower costal margin, and hip circumference was measured on the widest part around the greater trochanters. The waist-to-hip ratio (WHR) was calculated by waist circumference divided by hip circumference [32]. The percentage of body fat was assessed using an impedance meter (Tanita, Tokyo, Japan).

#### 2.3.3. Biological Parameters

Blood pressure was measured by a nurse using an electronic sphygmomanometer (Omron M3, HEM-7154-E) in female participants seated in a chair after a 15 min rest. Measurements were taken three times on both arms at 5 min rest intervals, and the mean value of the last two measurements was calculated and used for analysis.

Participants were asked to come in fasting from a 12 h overnight fasting period. Blood samples were obtained from the medial ulnar vein by a nurse between 9 and 10:30 am. The collected blood samples were used for the determination of plasma concentrations of fasting blood glucose and serum lipids, including, triglycerides, total cholesterol, LDL-cholesterol, and HDL-cholesterol. The blood samples were measured by the laboratory technicians of the hospital-Ksar el Kebir, and the results of the blood tests were subsequently retrieved on file.

#### 2.3.4. Physical Activity

To assess PA levels, we used the short version of the International PA Questionnaire (IPAQ-SF). This is a self-administered questionnaire that consists of assessing PA levels in adults aged 18–69 years, and its validity has been verified and confirmed in several countries [33]. The IPAQ-SF assesses, during the last seven days, the frequency (in days per week), and duration (in minutes per day) of total PA (only activities lasting more than 10 min without interruption were considered), namely walking and activities of moderate and vigorous intensity. Sedentary activity, i.e., the average time spent sitting, is also measured by this questionnaire. To measure physical activity, participants responded and reported their activities only during the previous 7 days, including walking (any type of walking), moderate activities (carrying light loads, gardening, doubles tennis, and cycling at a steady pace), and vigorous activities (carrying heavy loads, aerobics, weight training, fast cycling, or jogging at 10 km/h).

The results of the measured PA (walking, moderate, and vigorous intensity activities) were converted into minutes per week, they were then multiplied by specific METs factors (estimated energy expenditure at rest) for each activity, which are presented as: 3.3 METs for walking, 4 METs for moderate PA and 8 METs for vigorous PA [34]. The results thus obtained were expressed in metabolic equivalent minutes per week (MET-min/week). The total PA score was calculated by summing the result of each given activity. Resting time in a seated position was not included in the assessment of PA level. According to the IPAQ scoring protocol, PA levels were classified into three categories: low PA (less than 600 MET-min/week), moderate PA (at least 600 MET-min/week), and intense/vigorous PA (at least 3000 MET-min/week) [34].

### 2.4. Diagnostic Criteria

Overweight and obesity were determined by BMI, according to World Health Organization (WHO) criteria [35]. Participants were in: Underweight if BMI < 18.5 (kg/m^2^); Normal weight if 18.50 < BMI < 24.99 (kg/m^2^); Overweight if 25 < BMI < 29.99 (kg/m^2^); Obese if BMI ≥ 30 (kg/m^2^). Abdominal obesity was estimated by a waist circumference greater than or equal to 80 cm, waist to hip circumference ratio (WHR) greater than 0.85 cm [36]. High blood pressure was defined in participants with a systolic blood pressure (SBP) greater than or equal to 140 mmHg, and/or a diastolic blood pressure greater than or equal to 90 mmHg [37].

According to WHO criteria, type 2 diabetes was diagnosed as fasting blood glucose greater than or equal to 1.26 g/L, and pre-diabetes was diagnosed as fasting blood glucose between 1.10 and 1.25 g/L [38]. The diagnosis of hyperlipidemia was based on the National Cholesterol Education Program Adult Treatment Panel III (NCEP-ATP III) criteria. It was defined as: triglycerides ≥ 2.00 g/L, total cholesterol ≥ 2.40 g/L, HDL-cholesterol < 0.40 g/L, and/or LDL-cholesterol ≥ 1.60 g/L [39].

Metabolic syndrome was also defined according to the criteria of the NCEP-ATP III definition [40], by the presence of at least three out of five of the following risk factors: waist circumference (WC) > 88 cm, triglycerides (TG) ≥ 1.50 g/L, HDL-cholesterol (HDL-C) < 0.50 g/L, systolic blood pressure ≥ 130 mmHg, diastolic blood pressure ≥ 85 mmHg, and fasting blood glucose ≥ 1.10 g/L.

### 2.5. Statistical Analysis

For statistical analysis, we used the Statistical Package for Social Sciences (SPSS) software version 26.0. Quantitative variables were presented as mean ± standard deviation and qualitative variables were presented using numbers and percentages. The Chi-square test was used to assess the comparison of categorical variables, and the nonparametric Mann–Whitney and Kruskal–Wallis tests were used to compare continuous variables. Pearson correlations were used to investigate the association between physical activity, anthropometric and biological parameters of body composition, and metabolic disorders. For the entire analysis, *p* value < 0.05 was considered statistically significant.

## 3. Results

The study population is composed of 373 postmenopausal women, of which, 160 women were perimenopausal (42.9%) and 213 women were postmenopausal (57.1%). According to IPAQ guidelines, the average PA score of perimenopausal women was 1683.51 ± 805.36 MET-min/week, which was higher than that of postmenopausal women, at 1450.81 ± 780.67 MET-min/week, with a statistically significant difference (*p* < 0.05). According to IPAQ scores, perimenopausal participants were more physically active [moderate PA (84.37%) and intense PA (11.25%)] than postmenopausal participants [moderate PA (80.75%) and intense PA (7.51%)] (Table 1).

As shown in Table 2, in peri- and postmenopausal women, there were significant differences between PA levels and anthropometric parameters (weight, BMI, waist circumference, hip circumference, and percent body fat) (*p* < 0.001), and between biological parameters (fasting blood glucose (*p* < 0.002), total cholesterol, triglycerides, and LDL-C) (*p* < 0.001). No statistically significant difference was detected between PA levels and between HDL-C, systolic and diastolic blood pressures. There was a significant difference between PA levels and age in postmenopausal women (*p* < 0.05).

All participants with a low PA level had significantly higher mean values of anthropometric parameters than participants with moderate and intense PA levels, including weight, BMI, WC, HC, and body fat (%). The means of fasting blood glucose, total cholesterol, triglycerides, and LDL-C were also higher in participants with a low PA level than in those with moderate and intense PA levels. The majority of women in our study have moderate PA, all mean parameters except for HDL-C levels were higher for postmenopausal women than perimenopausal women, according to moderate PA level.

The frequencies of metabolic disorders, including overweight, obesity, body fat (obese), abdominal obesity, hypertension, pre-diabetes, diabetes, and MetS, were higher in peri- and postmenopausal women with a low level of PA than in those with moderate and intense PA levels.

In the moderate PA group, 36% of postmenopausal women were overweight, 58.8% were obese, 52.9% had an obese level of body fat, and more than 95% had abdominal obesity; those in perimenopause had frequencies of: 34.1% overweight, 17.8% obese, 47.4% with an obese level of body fat, and more than 97% with abdominal obesity. Regarding intense PA level, more than 50% of postmenopausal participants were overweight (37.5%) and obese (25%), and more than 30% of perimenopausal participants were overweight (5.5%) and obese (27.8%).

Perimenopausal women with moderate and intense PA levels had lower frequencies of hypertension (20%), diabetes (34.8%), and MetS (49.6%) than postmenopausal women (hypertension (34.3%), diabetes (43.6%), and MetS (72.1%)). In postmenopausal participants with a low PA level, the frequencies of elevated serum lipid concentrations (TC, TG, LDL-C) were lower than in perimenopausal participants; conversely, they were higher in postmenopausal participants than in perimenopausal participants with moderate and intense PA levels. There was a statistically significant difference between PA levels and the number of MetS components (*p* < 0.002); indeed, the percentages of women who had 4 or 5 MetS components were lower at moderate and intense PA levels, respectively (Table 3).

In the perimenopausal group, significant negative correlations were found between PA score (IPAQ) and weight (r = −0.341, *p* < 0.01), BMI (r = −0.402, *p* < 0.01), waist circumference (r = −0.333, *p* < 0.01), hip circumference (r = −0. 294, *p* < 0.01), percent body fat (%) (r = −0.301, *p* < 0.01), fasting blood glucose (r = −0.214, *p* < 0.01), total cholesterol (r = −0. 369, *p* < 0.01), triglycerides (r = −0.367, *p* < 0.01), LDL-C (r = −0.356, *p* < 0.01), and number of MetS components (r = −0.345, *p* < 0.01), respectively.

In the postmenopausal group, significant negative correlations were observed between PA score IPAQ and age (r = −0.192, *p* < 0.01), weight (r = −0.336, *p* < 0.01), BMI (r = −0.339, *p* < 0.01), waist circumference (r = −0.301, *p* < 0.01), hip circumference (r = −0.320, *p* < 0.01), percent body fat (%) (r = −0.346, *p* < 0.01), fasting blood glucose (r = −0.141, *p* < 0.05), total cholesterol (r = −0.277, *p* < 0.01), triglycerides (r = −0.137, *p* < 0.05), LDL-C (r = −0.297, *p* < 0.01), and number of MetS components (r = −0.200, *p* < 0.01), respectively.

No significant correlation was revealed between the PA score IPAQ and SBP, DBP, WHR, and HDL-C, respectively, in either group of women (Table 4).

## 4. Discussion

In the present study, we assessed PA levels using the IPAQ-SF questionnaire and investigated the association between PA, body composition, and metabolic disorders using anthropometric and biological parameters in middle-aged, peri- and postmenopausal women from the city of Ksar el Kebir. With the exception of WHR, SBP, DBP, and HDL-C, all mean parameters, including the number of MetS components, were significantly and inversely associated with PA. Participants with a low level of PA showed disturbing results, whereas participants with moderate and intense levels of PA had more favorable body composition and fewer metabolic disorders, including obesity, abdominal obesity, diabetes, dyslipidemia, and MetS. Abdominal obesity was the most abundant metabolic disorder in our population. Nevertheless, healthier outcomes were observed more in perimenopausal women than in postmenopausal women.

In our study, the majority of perimenopausal (84.37%) and postmenopausal (80.75%) women were classified as active and reported a moderate PA level. Our results are in agreement with studies by Bondarev et al. [41] and Moilanen et al. [42] showing that most peri- and postmenopausal women had a moderate level of PA, in contrast to other studies where most women were classified as inactive [43,44]. El Hajj et al. showed in their study that perimenopausal women were more active than postmenopausal women [45], which is also consistent with our findings that perimenopausal participants were more physically active at both moderate and intense PA levels compared to postmenopausal participants.

The literature has reported that PA decreases with age [46]. At middle age [46], postmenopausal women tend to decrease their PA and decrease their fitness more than women during the menopausal transition [47]. Our results agree with the literature. Indeed, in postmenopausal women, age was significantly associated with PA, and those with a low PA level were the oldest in the study population, with a mean age of 59.16 ± 3.77 years, height the mean age of 55.3 ± 2.8 years of postmenopausal women with a low PA level, reported by Lwow et al. [48]. Perimenopausal women with a low PA level were also older than physically active women. In a study from Nigeria, postmenopausal women with a low PA level were older than those who were active [49]. Studies have shown that reducing sedentary behavior [20], and hormone replacement therapy [47], can be used to maintain PA level and improve physical capacity in postmenopausal women. Regular PA practice and a balanced diet contribute to the prevention of obesity [6] and, thus, can contribute to the maintenance of sufficient PA level.

The results of the present study showed that anthropometric parameters were significantly and inversely associated with PA (*p* < 0.01); thus, the mean values of these parameters were significantly lower in women with moderate and intense levels of PA than in those at lower PA levels. Results similar to ours have also indicated that anthropometric parameters are associated with PA, and are higher at low PA [50,51]. Nevertheless, perimenopausal women who were physically active at moderate and intense levels had significantly lower of weight, BMI, body fat (%), HC, and WC compared with postmenopausal women. Indeed, more favorable body composition was observed at moderate and intense levels of PA in both peri- and postmenopausal women. Previous studies have shown that the frequencies of abdominal obesity and obesity were significantly lower in physically active middle-aged women [43,52]. Other studies have assessed PA in middle-aged women using a pedometer measuring steps per day, and reported that PA is inversely associated with BMI, WC, and body fat [53,54]. In this study, the incidence of obesity was significantly lower in women with moderate and intense PA levels, and a very low frequency was observed in moderately active perimenopausal women. Although there was a negative association between PA and WC, abdominal obesity, as determined by WC as an indicator, was significantly prevalent in both peri- and postmenopausal women (more than 90%) at all PA levels; however, the frequency of abdominal obesity appeared to be slightly lower in physically active women (moderate and intense PA levels). This finding may be due to insufficient PA and increased visceral fat accumulation that may be explained by the loss of estrogen resulting from the change in hormone secretion that peri- and postmenopausal women undergo during this critical period.

Although our results showed no statistically significant association between PA, SBP, and DBP, the frequency of hypertension (HAT) was lower in participants characterized by moderate and intense PA levels. Ogwumike et al. found the same results in their study [49], in contrast to other researchers who found a significant association between PA and blood pressure [55]. Yet, as the association between PA and blood pressure is somewhat debated, future studies may be needed to verify inconsistent results.

In this study, significant negative correlations were found between PA, FBG, and serum lipid concentrations (TC, TG, and LDL-C) (*p* < 0.05). We also observed significant differences between diabetes, dyslipidemia, and PA levels (*p* < 0.002). Participants with low PA had the highest FBG means, and were the most diabetic, and also had elevated TC, TG, and LDL-C concentrations characterizing a high frequency of dyslipidemia. Colpani et al. [43] and Lwow et al. [48] reported no significant relationship between PA and serum lipids, which is inconsistent with our results. In contrast, the results found by Woolf et al. were close to ours [51]. Similar results were observed in a study conducted in Egypt, assessing PA in postmenopausal women using the IPAQ, showing that diabetes was significantly less frequent at moderate and intense levels of PA [23]. Mainous et al. showed that a low PA level is associated with abnormal FBG and that PA is associated with the prevention and treatment of pre-diabetes and diabetes [56]. Some evidence has reported that menopause is associated with a higher risk of type 2 diabetes [15], and that abdominal obesity is a strong predictor of type 2 diabetes and impaired glucose tolerance, which may be observed mainly in older women [46]. However, PA has also been shown to be inversely associated with the prevalence and incidence of type 2 diabetes [46]. In addition, certain types of aerobic and resistance exercise are associated with a decreased risk of diabetes [17]. An unexpected result was found in our study: despite abdominal obesity being very common in peri- and postmenopausal women with moderate and intense PA levels, the frequency of diabetes was observed in less than 45% of these women. In addition, normal and healthy FBG concentrations were observed in perimenopausal (0.96 ± 0.35 g/L) and postmenopausal (1.08 ± 0.4 g/L) women with an intense PA level.

During menopause, dyslipidemia is characterized by an increase in TC, TG, and LDL-C levels [13], and these mainly in postmenopausal women rather than perimenopausal [14]. Researchers have reported that the impact of PA on serum lipids is stronger when accompanied by weight loss [21]. Other researchers reported in a recent study that PA contributes to a healthy lipid profile in menopausal women, and that PA measured by self-report or accelerometer is associated with lower blood lipid levels [57]. This is observed in our results, with the exception of HDL-C, mean TC, TG, and LDL-C concentrations were significantly lower in women who had moderate and intense levels of PA. Moreover, we found that at these PA levels, these mean concentrations were normal and healthy values in both peri- and postmenopausal women. Interestingly, we found no significant association between PA and HDL-C. Furthermore, results on HDL-C levels during menopause are still contradictory [30,57]. Furthermore, in previous studies, the results found on HDL-C levels and PA are also inconsistent: some researchers found that they increase with PA [58], while Owens et al. showed that these levels decrease in physically active women [59]. Clearly, further longitudinal studies are needed to clarify these findings.

In addition, our results showed a significant inverse association between PA and the number of MetS components defined according to NCEP-ATP III criteria (*p* < 0.01). This is consistent with the results found in a study conducted on Iranian adults [60]. In comparison with our results, the study by Hyvärinen et al. showed no association between PA and MetS components in menopausal women [57]. The highest percentage of MetS 3-component clustering was observed in peri- and postmenopausal women with an intense PA level, whereas that of the 4- and 5-component clustering was observed in women with a low PA level. The results of a recent study of Korean menopausal women are close to ours, showing that a large percentage of physically active women had 3-components, and a large percentage of inactive women had 4-or even 5-MetS components [61].

Menopause is considered a predictor of MetS [62]. It has been shown that during menopause, the prevalence of MetS and the clustering of its components tend to increase [15], and obesity largely leads to an increased prevalence of MetS [63]. In addition, abdominal obesity is a major cause of insulin resistance and MetS occurrence, and is positively associated with high MetS prevalence during menopause [30]. However, MetS prevalence is also associated with low PA and exercise capacity [64]. Aparicio et al. reported in their study of perimenopausal Moroccan women that low fitness increases the risk of having MetS and suggested including fitness as a risk factor for MetS [28]. Different research has established that PA is associated with a decrease in the number of MetS components, an improvement in each component, and a decrease in the incidence and risk of developing MetS [65,66]. Resistance training has been established to have a role in the prevention of MetS [21]. Regular aerobic PA also decreases the risk of developing MetS [64]. Other researchers have shown that low-intensity and less structured PA [65] and walking as PA three to five times per week [67] may decrease the incidence and probability of developing MetS. In this study, as mentioned above, there were significant negative correlations between PA and three of the five components of MetS: WC, TG, and FBG. The frequency of MetS was significantly lower in women with moderate and intense PA levels, compared with the frequency of MetS in women with a low PA level, which was very high (100% in perimenopause and 96% in post-menopause). Similar results to ours have been reported in studies also assessing PA and MetS using a self-administered questionnaire, and the NCEP-ATP III definition, respectively [50,60]. We found that dyslipidemia, diabetes, and MetS were less common in perimenopausal women than in postmenopausal women reporting moderate and intense levels of PA.

However, our study has limitations that should be noted: (1) the cross-sectional design of this study that precludes the determination of causality; (2) the small sample size, and ethnic and cultural homogeneity that preclude extrapolation of the results to more heterogeneous populations; (3) the assessment of PA being based on a self-reported questionnaire (IPAQ-SF), which could influence the classification of participants according to PA levels; and (4) menopausal status being determined with a self-reported menstrual diary and not with serum hormone measurements, meaning the study lacked accurate information on menopausal status. Other limitations were also identified: seasonal variations affecting PA, lack of dietary intake assessment, exclusion of hormone therapy, and menopause due to surgery. In the future, studies with a large representative sample will be of great value in providing results that can be generalized to other, heterogeneous populations.

## 5. Conclusions

In conclusion, our results indicate that moderate and intense levels of PA were associated with more favorable body composition and less frequent metabolic disorders compared with a low PA level in middle-aged, peri- and postmenopausal women. However, perimenopausal women showed healthier outcomes than postmenopausal women. Although this study showed significantly inverse associations between PA and most parameters of body composition, and metabolic disorders in peri- and postmenopausal women, it did not show significant associations between PA, blood pressure, and HDL-C. Therefore, further and more extensive longitudinal studies on the role of PA in the general health of menopausal women in Morocco are needed.

## Figures and Tables

**Table 1:** Total score and physical activity levels of the study population According to the IPAQ.

Perimenopause (*n* = 160)			PostMenopause (*n* = 213)		
	*n* (%)	Mean ± SD (MET-min/Week)	*n* (%)	Mean ± SD (MET-min/Week)	*p*-Value
Low PA	7 (4.38)	498.86 ± 51.89	25 (11.74)	460.82 ± 118.62	0.927
Moderate PA	135 (84.37)	1517.6 3 ± 505.46	172 (80.75)	1416.07 ± 502.33	0.045 *
Intense PA	18 (11.25)	3388.28 ± 421.85	16 (7.51)	3371.29 ± 364.86	0.717
Total Score IPAQ	160	1683.51 ± 805.36	213	1450.81 ± 780.67	0.001 *

PA = Physical activity; MET-min/week = metabolic equivalent- minutes per week. Values are presented as mean ± standard deviation and by number and percentage in parentheses. * Significant: *p* < 0.05.

**Table 2:** Anthropometric and biological parameters of the study classified by physical activity level.

	Physical Activity (IPAQ)							
	Perimenopause (*n* = 160)				PostMenopause (*n* = 213)			
PA Levels	Low	Moderate	Intense	*p*-Value	Low	Moderate	Intense	*p*-Value
Age (years)	49.43 ± 3.05	48.84 ± 2.44	48.61 ± 1.94	0.917	59.16 ± 3.77	56.48 ± 4.30	54.63 ± 3.36	0.001 *
Age group, *n* (%)								
45–49	4 (57.1)	84 (62.2)	10 (55.6)	0.926	1 (4)	11 (6.4)	0 (0)	0.003 *
50–54	3 (42.9)	47 (34.8)	7 (38.9)		2 (8)	47 (27.3)	10 (62.5)	
55–59	0 (0)	4 (3)	1 (5.6)		8 (32)	61 (35.5)	5 (31.3)	
60–64	NA	NA	NA		14 (56)	53 (30.8)	1 (6.2)	
***Anthropometry, Body composition***								
Weight (kg)	95.76 ± 10.98	76.22 ± 11.8	66.11 ± 7.06	0.000 *	94.22 ± 18.5	78.78 ± 13.53	67.02 ± 10.29	0.000 *
BMI (kg/m^2^)	39.88 ± 3.7	30.08 ± 4.29	26.56 ± 2.33	0.000 *	37.17 ± 6.44	31.58 ± 4.92	26.95 ± 3.69	0.000 *
WC (cm)	115.43 ± 11.17	99.16 ± 9.96	92.5 ± 6.33	0.000 *	114.64 ± 13.06	104.03 ± 10.4	95 ± 9.75	0.000 *
HC (cm)	119.71 ± 10.17	106.3 ± 9.62	99.94 ± 5.26	0.000 *	120.88 ± 12.77	108.72 ± 10.15	102.19 ± 10.89	0.000 *
Body fat (%)	47.23 ± 1.89	39.88 ± 5.5	35.01 ± 6.14	0.000 *	45.45 ± 5.22	41.58 ± 5.42	34.63 ± 6.3	0.000 *
***Biological parameters***								
SBP (mmHg)	141.57 ± 22.63	127.12 ± 16.07	125.44 ± 13.87	0.223	138.8 ± 13.33	131.08 ± 16.22	133 ± 18.53	0.053
DBP (mmHg)	85.14 ± 21.28	76.11 ± 10.77	75.22 ± 14.48	0.635	82.68 ± 10.11	78.22 ± 11.47	76.81 ± 13.66	0.057
FBG (g/L)	1.52 ± 0.46	1.21 ± 0.5	0.96 ± 0.35	0.001 *	1.69 ± 0.59	1.31 ± 0.52	1.08 ± 0.4	0.000 *
TC (g/L)	3.00 ± 0.53	1.67 ± 0.5	1.38 ± 0.15	0.000 *	2.68 ± 0.71	1.91 ± 0.62	1.53 ± 0.54	0.000 *
TG (g/L)	2.15 ± 0.55	1.02 ± 0.46	0.76 ± 0.18	0.000 *	1.91 ± 0.62	1.3 ± 0.66	1.00 ± 0.58	0.000 *
LDL-C (g/L)	2.12 ± 0.37	1.01 ± 0.41	0.81 ± 0.15	0.000 *	1.81 ± 0.55	1.19 ± 0.5	0.87 ± 0.47	0.000 *
HDL-C (g/L)	0.41 ± 0.06	0.43 ± 0.08	0.39 ± 0.07	0.102	0.43 ± 0.06	0.42 ± 0.06	0.42 ± 0.08	0.893

PA = Physical activity; BMI = Body Mass Index; WC = Waist circumference; HC = Hip circumference; SBP = Systolic blood pressure; DBP = Diastolic blood pressure; FBG = Fasting blood glucose; TC = Total cholesterol; TG = Triglycerides; LDL-C = Low Density Lipoproteins-Cholesterol; HDL-C = High Density Lipoproteins-Cholesterol; NA = Not applicable. Values are presented as mean ± standard deviation, and by number and percentage in parentheses. * Significant: *p* < 0.05.

**Table 3:** Frequency of metabolic disorders in peri- and postmenopausal participants according to physical activity level.

	Physical Activity (IPAQ)							
	Perimenopause (*n* = 160)				PostMenopause (*n* = 213)			
PA Levels	Low	Moderate	Intense	*p*-Value	Low	Moderate	Intense	*p*-Value
***Overweight, obesity, n (%)***								
BMI, *n* (%)								
Underweight	NA	NA	NA	0.000 *	0 (0)	1 (0.5)	0 (0)	0.000 *
Normal weight	0 (0)	65 (48.1)	12 (66.7)		1 (4)	8 (4.7)	6 (37.5)	
Overweight	2 (28.6)	46 (34.1)	1 (5.5)		2 (8)	62 (36)	6 (37.5)	
Obesity	5 (71.4)	24 (17.8)	5 (27.8)		22 (88)	101 (58.8)	4 (25)	
***Body fat, n (%)***								
Underfat	0 (0)	1 (0.7)	2 (11.1)	0.000 *	0 (0)	2 (1.2)	1 (6.3)	0.000 *
Healthy	0 (0)	17 (12.6)	5 (27.8)		1 (4)	17 (9.9)	7 (43.8)	
Overfat	0 (0)	53 (39.3)	10 (55.6)		7 (28)	62 (36)	6 (37.5)	
Obese	7 (100)	64 (47.4)	1 (5.5)		17 (68)	91 (52.9)	2 (12.4)	
***Abdominal obesity, n (%)***								
WC, *n* (%)								
Normal	0 (0)	3 (2.2)	1 (5.6)	0.000 *	0 (0)	1 (0.6)	1 (6.3)	0.000 *
Abdominale	7 (100)	132 (97.8)	17 (94.4)		25 (100)	171 (99.4)	15 (93.7)	
obesity								
***WHR* *, n (%)***								
Normal	0 (0)	1 (0.7)	0 (0)	0.001 *	0 (0)	1 (0.6)	0 (0)	0.001 *
High	7 (100)	134 (99.3)	18 (100)		25 (100)	171 (99.4)	16 (100)	
***Blood pressure (HTA), n (%)***								
Normal	3 (42.8)	108 (80)	15 (83.3)	0.887	13 (52)	113 (65.7)	11 (68.8)	0.449
High BP	4 (57.2)	27 (20)	3 (16.7)		12 (48)	59 (34.3)	5 (31.2)	
***Diabetes type 2, n (%)***								
Non-diabetics	1 (14.3)	87 (64.4)	16 (88.9)	0.000 *	4 (16)	94 (54.7)	12 (75)	0.001 *
Pre-diabetes	1 (14.3)	1 (0.8)	0 (0)		2 (8)	3 (1.7)	0 (0)	
Diabetes	5 (71.4)	47 (34.8)	2 (11.1)		19 (76)	75 (43.6)	4 (25)	
***Dyslipidemia, n (%)***								
TC, *n* (%)								
Normal	0 (0)	113 (83.7)	18 (100)	0.000 *	6 (24)	116 (67.4)	14 (87.5)	0.000 *
High	7 (100)	22 (16.3)	0 (0)		16 (76)	56 (32.6)	2 (12.5)	
TG, *n* (%)								
Normal	1 (14.3)	119 (88.1)	18 (100)	0.000 *	7 (28)	113 (65.7)	14 (87.5)	0.001 *
High	6 (85.7)	16 (11.9)	0 (0)		18 (72)	59 (34.3)	2 (12.5)	
LDL-C, *n* (%)								
Normal	0 (0)	87 (64.4)	17 (94.4)	0.000 *	2 (8)	90 (52.3)	13 (81.1)	0.000 *
High	7 (100)	48 (35.6)	1 (5.6)		23 (92)	82 (47.7)	3 (18.9)	
HDL-C, n (%)								
Low	0 (0)	1 (0.7)	0 (0)	0.886	0 (0)	1 (0.6)	1 (6.2)	0.302
Normal	0 (0)	5 (3.7)	0 (0)		0 (0)	9 (5.2)	4 (25)	
High	7 (100)	129 (95.6)	18 (100)		25 (100)	162 (94.2)	11 (68.8)	
***Metabolic syndrome, n (%)***								
−MetS	0 (0)	68 (50.4)	11 (61.1)	0.020 *	1 (4)	48 (27.9)	6 (37.5)	0.021 *
+MetS	7 (100)	67 (49.6)	7 (38.9)		24 (96)	124 (72.1)	10 (62.5)	
Number of MetS components, n (%)								
3 components	2 (28.6)	35 (25.9)	7 (38.9)	0.001 *	4 (16)	44 (25.6)	8 (50)	0.000 *
4 components	2 (28.6)	29 (21.5)	0 (0)		7 (28)	46 (26.7)	0(0)	
5 components	3 (42.9)	3 (2.2)	0 (0)		13 (52)	34 (19.8)	2 (12.5)	
mean ± SD	4.14 ± 0.90	2.58 ± 1.10	2.17 ± 0.79	0.001 *	4.24 ± 1.01	3.32 ± 1.20	2.56 ± 1.32	0.000 *

PA = Physical activity; BMI = Body Mass Index; WC = Waist circumference; WHR = Waist to Hip Ratio; HTA = Hypertension; BP = Blood pressure; TC = Total cholesterol; TG = Triglycerides; LDL-C = Low Density Lipoproteins-Cholesterol; HDL-C = High Density Lipoproteins-Cholesterol; MetS = Metabolic syndrome; −MetS = Absence of Metabolic syndrome; +MetS = Presence of Metabolic syndrome. Values are presented as mean ± standard deviation, and by number and percentage in parentheses. * Significant: *p* < 0.05.

**Table 4:** Correlations between anthropometric and biological study parameters, and physical activity (IPAQ) in peri- and postmenopausal women.

	Score-IPAQ (MET-min/Week)			
	Perimenopause (*n* = 160)		PostMenopause (*n* = 213)	
	r-Value	*p*-Value	r-Value	*p*-Value
Age (years)	0.006	0.937	−0.192	0.005 **
Weight (kg)	−0.341	0.000 **	−0.336	0.000 **
BMI (kg/m^2^)	−0.402	0.000 **	−0.339	0.000 **
Waist circumference (cm)	−0.333	0.000 **	−0.301	0.000 **
Hip circumference (cm)	−0.294	0.000 **	−0.320	0.000 **
WHR	−0.111	0.163	0.013	0.851
Body fat (%)	−0.301	0.000 **	−0.346	0.000 **
SBP (mmHg)	−0.079	0.318	−0.016	0.819
DBP (mmHg)	−0.001	0.994	−0.008	0.910
Fasting blood glucose (g/l)	−0.214	0.007 **	−0.141	0.039 *
Total cholesterol (g/L)	−0.369	0.000 **	−0.277	0.000 **
Triglycerides (g/L)	−0.367	0.000 **	−0.137	0.045 *
LDL-C (g/L)	−0.356	0.000 **	−0.297	0.000 **
HDL-C (g/L)	−0.057	0.472	−0.075	0.275
Number of MetS components	−0.345	0.000 **	−0.200	0.003 **

IPAQ = International PA Questionnaire; MET-min/week = metabolic equivalent-minutes per week; BMI = Body Mass Index; WHR = Waist to Hip Ratio; SBP = Systolic blood pressure; DBP = Diastolic blood pressure; LDL-C = Low Density Lipoproteins-Cholesterol; HDL-C = High Density Lipoproteins-Cholesterol; MetS = Metabolic syndrome. ** Significative: *p* < 0.01. * Significative: *p* < 0.05.

## Data Availability

Not applicable.
